# Repetitive TMS on Left Cerebellum Affects Impulsivity in Borderline Personality Disorder: A Pilot Study

**DOI:** 10.3389/fnhum.2016.00582

**Published:** 2016-12-05

**Authors:** Giulia Zelda De Vidovich, Riccardo Muffatti, Jessica Monaco, Nicoletta Caramia, Davide Broglia, Edgardo Caverzasi, Francesco Barale, Egidio D’Angelo

**Affiliations:** ^1^Department of Brain and Behavioral Sciences, University of PaviaPavia, Italy; ^2^Psychiatry Unit, Santi Paolo e Carlo Hospital of MilanMilan, Italy; ^3^Interdepartmental Center for Research on Personality Disorders, University of PaviaPavia, Italy; ^4^Brain Connectivity Center, C. Mondino National Neurological InstitutePavia, Italy

**Keywords:** TMS, cerebellum, borderline personality disorder, impulsivity, Go/No-Go task

## Abstract

The *borderline personality disorder* (BPD) is characterized by a severe pattern of instability in emotional regulation, interpersonal relationships, identity and impulse control. These functions are related to the prefrontal cortex (PFC), and since PFC shows a rich anatomical connectivity with the cerebellum, the functionality of the cerebellar-PFC axis may impact on BPD. In this study, we investigated the potential involvement of cerebello-thalamo-cortical connections in impulsive reactions through a pre/post stimulation design. BPD patients (*n* = 8) and healthy controls (HC; *n* = 9) performed an Affective Go/No-Go task (AGN) assessing information processing biases for positive and negative stimuli before and after repetitive transcranial magnetic stimulation (rTMS; 1 Hz/10 min, 80% resting motor threshold (RMT) over the left lateral cerebellum. The AGN task consisted of four blocks requiring associative capacities of increasing complexity. BPD patients performed significantly worse than the HC, especially when cognitive demands were high (third and fourth block), but their performance approached that of HC after rTMS (rTMS was almost ineffective in HC). The more evident effect of rTMS in complex associative tasks might have occurred since the cerebellum is deeply involved in integration and coordination of different stimuli. We hypothesize that in BPD patients, cerebello-thalamo-cortical communication is altered, resulting in emotional dysregulation and disturbed impulse control. The rTMS over the left cerebellum might have interfered with existing functional connections exerting a facilitating effect on PFC control.

## Introduction

The *borderline personality disorder* (BPD) is a complex and serious mental disorder characterized by a pervasive pattern of instability in affective regulation, interpersonal relationships, self image and behavioral control (Siever et al., 2002; Skodol et al., 2002a,b). BPD is characterized by progressive functional impairment, substantial treatment utilization and a mortality rate by suicide of almost 10–50 times higher than the rate in the general population (American Psychiatric Association, 2001).

The median population prevalence is estimated to be 1.6% but may be as high as 5.9%. The prevalence of BPD is about 6% in primary care settings, about 10% among individuals seen in outpatient mental health clinics, and about 20% among psychiatric inpatients (DSM-5). The prevalence of BPD may decrease in older age groups (DSM-5). Impulsivity and aggressiveness are heritable traits that may contribute to the psychopathology of BPD (Lieb et al., 2004; Siever and Weinstein, 2009).

Impulsive behavior has been conceptualized as an imbalance between the “top-down” control or “brakes” provided by the frontal cortices and excessive “bottom-up drives” triggered or signaled by limbic regions (Siever, 2008). Regarding prefrontal cortices, the dorsolateral prefrontal cortex (DLPFC) represents a key structure, along with the orbitofrontal cortex, in impulsivity control (Soloff et al., 2003; Chanen et al., 2008; Matsuo et al., 2009). Reduction of DLPFC gray matter volumes has recently been shown in BPD patients compared to healthy controls (HC; Tomoda et al., 2009; Brunner et al., 2010). Moreover, a recent PET study on BPD patients, showed that DLPFC failed to activate during the top-down cognitive control of aggression (New et al., 2007, 2009). Recent meta-analysis (Ruocco et al., 2013; Schulze et al., 2016) evidenced bilaterally reduced activation within the DLPFC in BPD patients compared to healthy subjects associated with negative emotion processing, thus confirming the PFC’s role in emotion regulation through altered connections with the insula. Finally, the interplay between DLPFC and hippocampus appears to be deeply involved memory, emotional and behavioral control in BPD (Schmahl et al., 2003, 2004; Anderson et al., 2004; New et al., 2007; Sala et al., 2009; Morandotti et al., 2013).

Literature about prefrontal functions in BPD has been controversial for many years and the cognitive profile of BPD remains to be fully clarified. Clinical theoreticians and researchers have proposed that the psychopathology of BPD is associated with disruptions in basic neurocognitive processes. BPD patients perform worse than HC in multiple neurocognitive domains (Bazanis et al., 2002; Dinn et al., 2004; Monarch et al., 2004). Other empirical evidences are inconsistent with these results and revealed a lack of specificity and stability of these reported neuropsychological deficits (Fertuck et al., 2005; LeGris and van Reekum, 2006; Silbersweig et al., 2007). A recent meta-analysis (Unoka and Richman, 2016) however highlighted impairments in neuropsychological functioning in BPD, especially in domains of decision making, memory, executive functioning, processing speed, verbal intelligence and visuospatial abilities. Moreover BPD seem to perform differently according to specific inhibitory tasks. Behavioral inhibition focuses on holding or suppression of an already selected or initiated response and thus, late control processes (Stahl et al., 2014). Stop-signal and go/no-go tasks represent the most important behavioral inhibition tasks (Aron, 2011; Swick et al., 2011). “Experimental paradigms assessing emotionally neutral impulse control in BPD have revealed inconsistent results” (Sebastian et al., 2013). Two fMRI studies focusing on behavioral inhibition in emotional context have revealed prefrontal dysfunction especially if modulated by negative emotions (Silbersweig et al., 2007; Jacob et al., 2013). For these reasons we set up a specific test that assesses information processing biases for positive and negative stimuli (see “Materials and Methods” Section).

Recent anatomical and clinical evidence has shown that the cerebellum, primarily considered a motor control structure, is also involved in higher cognitive functions (Ito, 1993, 2005, 2008; Ivry et al., 2002; Schmahmann, 2004; D’Angelo and Casali, 2012) and behavioral changes, such as impulsive behavior. Neuroanatomical research has shown that the cerebellum projects to the PFC through the ventrolateral thalamic nucleus (VL) including the mediodorsal thalamic nucleus (MD; Yamamoto et al., 1992; Middleton and Strick, 2001) and the reticular nucleus of the thalamus (RNT; Çavdar et al., 2002). High resolution tractography in humans *in vivo* has recently shown that about 40% of fiber tracts leaving the cerebellum through the superior cerebellar peduncle actually reach the PFC through the VL (Palesi et al., 2015). A fMRI study (Allen et al., 2005) demonstrated fluctuations in signal in the dentate nucleus correlated with fluctuations in cerebellar, thalamic, limbic, striatal, and cerebrocortical regions including parietal and frontal sites, with prominent coherence in DLPFC (areas 9 and 46 mainly). These anatomical and functional connections between the cerebellum and the PFC suggest that the cerebellum is involved in non-motor circuits. Additional evidence that the cerebellum plays a key role in higher-order cognitive functions and behavior comes from imaging studies altered cerebellar volumes in patients with neuropsychiatric diseases such as Attention-Deficit/Hyperactivity Disorder (ADHD) and schizophrenia (Roth and Saykin, 2004; Andreasen and Pierson, 2008; Thomann et al., 2009) and altered cerebellar metabolism in patients with obsessive compulsive disorder (Pujol et al., 2004; Nabeyama et al., 2008). Impulsivity is a core feature of these neuropsychiatric diseases (King et al., 2003). Although in the literature the reports describe increased impulsivity in BPD, the evidence about cerebellar involvement is limited and inconsistent.

In addition to neuroimaging, recently TMS has also been used to identify neural substrates of psychiatric disorders. Repetitive transcranial magnetic stimulation (rTMS) involves the delivery of trains of magnetic pulses to produce changes in cortical excitability that persist beyond the duration of the stimulus. The mechanisms through which these protocols alter local neural circuits are believed to involve processes similar to synaptic long-term potentiation (LTP) and long-term depression (LTD; Fitzgerald et al., 2003). One of the earliest rTMS studies (Pascual-Leone et al., 1994) demonstrated that impulse trains at high-frequency (>5 Hz) generally increased cortical excitability (as measured by the size of motor evokd potentials [MEPs]). These effects persisted for 3–4 min after the end of stimulation. On the contrary, rTMS at frequencies of 1 Hz or below generally decreases cortical excitability. Recent studies showed that rTMS caused 30% changes in cortical excitability persisting for about 30 min in the EEG (Thut and Pascual-Leone, 2010).

Theta-burst cerebellar rTMS has been successfully used to modulate motor timing (Del Olmo et al., 2007) and procedural learning (Torriero et al., 2011) with a reflexion on finger movement (Del Olmo et al., 2007), saccadic eye movements (Colnaghi et al., 2011), eye-blink classical conditioning (Monaco et al., 2014). These effects are likely to involve metaplasticty of cerebello-cortical connectivity, with relevant effects in progressive supranuclear palsy (Brusa et al., 2014) in dystonia (Koch et al., 2014), ataxia (Bonnì et al., 2014). Theta-burst cerebellar rTMS proved able to interfere with electrical excitablity of the PFC in a Go-No-Go task (Picazio et al., 2016). Conversely, cerebellar low-frequency rTMS increased facilitation in the primary motor cortex (M1; Oliveri et al., 2005). In the current work we reasoned that an excitatory impact on the PFC could have been exerted by cerebellar low-frequency rTMS and that this effect could impact on the Go-no-Go task execution in BPD patients.

The rTMS-induced changes in motor cortex excitability have been monitored in various studies in a pre-post stimulation design similar to this experimental study. Changes in cortical excitability pre vs. post-rTMS stimulation were interpreted as measures of rTMS-induced changes in synaptic plasticity (Fitzgerald et al., 2006). A recent TMS study (Cailhol et al., 2014) has investigated the effect of a high-frequency rTMS in 10 sessions delivered over the right DLPFC in 10 BPD patients. BPD in the rTMS group showed improvements in anger, affective instability and planning, thus confirming our hypothesis about prefrontal involvement in BPD patients’ dyscontrol. The cerebellar TMS was used in previous studies to test non-motor systems. Del Olmo and Minsk (Del Olmo et al., 2007; Minks et al., 2010) used rTMS (1 Hz, 600 pulses) over cerebellar hemispheres and studied its effect on the performance of a finger-tapping; Koch et al. (2007) studied the perception of time with TMS in two experiments; Desmond et al. (2005) tested whether disruption of the right superior cerebellum was able to impair verbal working memory performance. To the best of our knowledge there are no previous studies focusing on cerebellar TMS in personality disorders. Moreover, we had to evaluate the problem of functional cortical asymmetry in BPD, in order to define the target brain area of stimulation. Right cerebral involvement seems to be predominant in the neurobiology of the disorder (Irle et al., 2005; Sala et al., 2009; Morandotti et al., 2013). In a previous study (Barnow et al., 2009), a reduced cortical silent period (CSP) in the right cortex was found in BPD patients compared with HC. Irle et al. (2005) supported a reduced CSP in the right cerebral cortex, but not in the left side, showing a reduced right parietal cortex volume in patients with BPD. The authors hypothesized a neurodevelopmental deficit in the right hemisphere that may be linked to several traumatic life events of BDP patients. Additionally, authors observed an increased leftward asymmetry as a protective factor in BPD patients with post-traumatic stress disorder (PTSD) for the development of disabling psychotic syndromes (Irle et al., 2005).

For these reasons we first focused on the circuit involving the left cerebellar cortex and the right PFC. The study should be extended in both cerebellar hemispheres in order to clarify the differences in cerebello-thalamo-cortical connections bilaterally. Cortical inhibition deficits have been demonstrated in several disorders with deficits in impulsive control (e.g., ADHD, tic disorder, Tourette syndrome) by using TMS protocol. Recent findings support an association between BPD and cortical inhibition deficits as evidenced through TMS (Barnow et al., 2009).

In this TMS study we aim at investigating whether cerebellum and PFC functions may be involved in impulsive reactions in BPD through a pre/post stimulation design.

The present study has been accepted by the local Ethical Commitee (11.09.2014) and received formal authorization from the Neurological Institute, Casimiro Mondino Foundation (17.10.2014).

## Materials and Methods

### Participants

In this pilot study eight DSM-IV BPD patients were recruited at the Center for Research on Personality Disorders of Pavia and at the Outpatient Service of San Paolo Hospital of Milan located in Rozzano District, Milan (mean age ± S.D. = 40 ± 10.7 years; four females; eight right-handed; years of education ± S.D. = 12.62 ± 2). The diagnosis was determined with the SCID-II (Williams et al., 1992) and successively confirmed with the clinical consensus of two psychiatrists. The SCID-I was administered in order to detect any Axis I disorders (Spitzer et al., 1992). The Zanarini Scale for Borderline Personality Disorders (ZAN-BPD; Zanarini et al., 2003) was used to rate the severity of the psychopathology. Handedness was detected with the Oldfield handedness questionnaire (Oldfield, 1971). Patients with any comorbid personality disorder, current medical problems, alcohol or substance abuse within 5 weeks preceding the study were excluded. Two patients were not taking any medication at the time of testing; three were treated with antidepressant and mood stabilizer, three were treated with a combination of antidepressant, mood stabilizers and antipsychotics. Three patients did not have any lifetime comorbid conditions, while two had major depression, and three patients had alcohol abuse in the past years.

- *Inclusion criteria*:
  Borderline Personality Disorder Diagnosis
  Age between18 and 45 years
  Right Hemispheric Dominance

- *Exclusion criteria*:
  Comorbid personality disorders
  Current medical problems
  Alcohol or substance abuse within 6 weeks preceding the study
  Epilepsy, seizure-like attacks, faint of unknown origin
  Pregnancy
  Pacemaker or metallic implants
  Years of education <8

Nine HC matched with eight BPD patients for race, gender, handedness were recruited from a departmental database of the University of Pavia (mean age ± S.D. = 31 ± 4 years; five females; nine right-handed; years of education = 17.8 ± 2). They had no past or current history of any axis I or II disorders as determined by the SCID non-patient version (SCID-NP), the SCID-II and the ZAN-BPD. Also they had no current medical problems, no history of substance/alcohol abuse, and no history of psychiatric disorders among first-degree relatives. The same scales as for the BPD patients were administered to HC.

The HRDS-24 (Hamilton, 1960) and the BPRS (Andersen et al., 1989) were used to rate psychiatric symptoms. To assess aggressive and impulsive behavior the Buss-Durkee Hostility Inventory (BDHI; Buss and Durkee, 1957) and the Barratt Impulsivity Scale (BIS-11; Patton et al., 1995) were, respectively, used. The Child Abuse Scale (CABUSE; Soloff et al., 2002) was utilized to evaluate childhood abuse and neglect experiences.

All the tests and rating scales were administered by trained raters with extensive experience, being fully reliable blindly and independently with a senior investigator.

All participants were naive to TMS at the beginning of the study. An informed consent was obtained from all participants, and the study was approved by the local Ethics Committee and conducted in accordance with regulations defined in the Declaration of Helsinki.

Each subject can decide to quit the study in every phase, independently from his/her medical treatment and therapeutic program.

### Neuropsychological Task

Neuropsychological evaluation consists of an Affective Go/No-go (AGN) task (Murphy et al., 1999). The test gives a measure of the ability and accuracy to enhance/inhibit a specific response. The subject has to maintain continuous attention, concentration and inhibitory control due to the frequent changements of the target. We set up a test with affective stimuli in order to evaluate if patients demonstrate impaired neuropsychological functions when dealing with negative semantic dimension. The AGN task assesses information processing biases for positive and negative stimuli. Stimuli are words belonging to different categories: positive emotions (e.g., Joyful, Ecstasy), negative emotions (e.g., Bad, Failure), fruits (e.g., Kiwi, Ananas) and insects (e.g., Cricket, Fly). Stimuli from the last two categories were chosen as examples of neutral (non emotional) stimuli. The AGN task consists of four experimental blocks. Each block is composed of 40 words, 10 for each category as shown in Table 1.

**Table 1:** **The 40 words used to generate the four experimental blocks (in *Italian* and English) are reported along with their category (POSITIVE, NEGATIVE, FRUITS, INSECTS)**.

*Italian POSITIVA*	English POSITIVA	*Italian NEGATIVA*	English NEGATIVA
*gioioso*	joyful	*amareggiato*	embittered
*felice*	happy	*noioso*	boring
*carino*	cute	*dispiaciuto*	regretful
*sorridente*	smiling	*frustrato*	frustrated
*allegro*	gleeful	*cattivo*	bad
*coraggioso*	courageous	*controproducente*	counterproductive
*estasi*	ecstasy	*cupo*	gloomy
*benefico*	beneficial	*fallimentare*	failure
*felice*	happy	*malinconico*	melancholy
*brillante*	brilliant	*deludente*	disappointing

**FRUTTA**	**FRUITS**	**INSETTO**	**INSECT**

*ananas*	pineapple	*grillo*	cricket
*pompelmo*	grapefruit	*mosca*	fly
*banana*	banana	*cicala*	cicada
*pera*	pear	*coleottero*	beetle
*kiwi*	kiwi	*ape*	bee
*ribes*	currant	*vespa*	wasp
*uva*	grapes	*formica*	ant
*mela*	apple	*coccinella*	ladybug
*arancia*	orange	*zanzara*	mosquito
*prugna*	plum	*cimice*	bug

The 40 words in a block are organized in two procedures (see below) and appear with a slide in the center of computer screen with random sequences. The subject has to respond in a specific time range (*ca.* 1000 ms) and correctly. A feedback display slide just after this time range appears to show if the response is correct or incorrect before the following slide. If the response is correct and within the set time limit, a blue circle appears below the word, otherwise a red cross indicates an error (i.e., incorrect or delayed response). The subject makes an error if he/she fails to make a correct response (CR) prior to the response deadline, set to be 1000 ms in the experiment, each block in AGN task is preceded by an instruction slide in order to show the target category we are seeking in this specific block. Participants are asked to press the space-bar when a word matching with the given target category appears. The blocks are organized in four blocks of increasing difficulty, as shown below.

The first block asks to choose the POSITIVE target category with two procedures: POSPOS shows 10 positive words and POSNEG shows 10 negative words, 10 fruits and 10 insects.

The second block asks to choose the NEGATIVE target category with two procedures: NEGNEG shows 10 negative words and NEGPOS shows 10 positive words, 10 fruits and 10 insects.

The third block asks to choose the POSITIVE-INSECT target category with two procedures: INS-POS-Correct shows 10 positive and 10 insects, whereas INS-POS-Incorrect, shows 10 fruits and 10 negative words.

The fourth block asks to choose the NEGATIVE-FRUITS target category with two procedures: FRU-NEG-Correct shows 10 fruits and 10 negative words, whereas FRU-NEG-Incorrect shows 10 insects and 10 positive words.

A complete example is reported in the link: http://www-5.unipv.it/dangelo/?page_id=4485.

### TMS Techniques

The TMS protocols were developed according to a previous work (Monaco et al., 2014). The resting motor threshold (RMT) was assessed in each participant by recording MEPs from the right first dorsal interosseous (FDI) muscle after applying single pulse TMS over the left M1. To this end, surface electrodes (Ag/AgCl) were placed over the muscle belly (i.e., active electrode) and over the corresponding tendon (i.e., reference) in a belly–tendon montage. The RMT was defined as the lowest stimulation intensity to evoke MEPs of at least 50 μV peak-to-peak in 5 out of 10 single pulse stimulations.

In the current study, we used a rTMS protocol which consists in a train of pulses delivered at frequency of 1 Hz and intensity of 80% of RMT for 10 min in order to obtain inhibitory effects.

We used a magnetic stimulator MagVenture (MagPro X100) with a double butterfly coil (mod. coil B-65). The TMS was applied over the left lateral cerebellum using the same scalp coordinates as in previous studies (1 cm inferior and 3 cm left to the inion; Théoret et al., 2001). These coordinates, adopted in previous magnetic resonance imaging studies, showed that this site targets the posterior lobules of the lateral cerebellum. The coil was positioned tangentially to the scalp (Koch et al., 2008) and the current in the coil was directed downward, in order to induce an upward current in the cerebellar cortex.

RMT was acquired with MP150 Biopac System. The AGN task was developed with E-prime.

### Experimental Procedures

The experimental procedure is the same in both groups and lasts approximately 90 min. Each subject was administered the AGN task twice, before and immediately after TMS protocol. Participants were comfortably seated on a chair in front of a 23-inch. computer screen in a quiet room with normal indoor lighting. The viewing distance was approximately 65 cm from the screen.

In order to avoid a significant learning effect between the first and the second neuropsychological task, subjects attend a short preliminary training (AGN short version with 10 words in each block) that has specific goals of improving one’s capability and skills. In the next step the AGN task assesses the information processing biases for positive and negative stimuli.

After the first AGN task, rTMS over the left lateral cerebellum is applied according to the rTMS protocol with details explained above.

Subjects’ performance in the AGN task is evaluated immediately after rTMS trains.

The final part of the experimental procedure is a clinical evaluation after 1 week aimed at collecting clinical global impressions and tolerability, subjective impressions reported by BPD patients.

Experimental procedures are conducted by a medical doctor and one biologist at the Brain Connectivity Center (BCC) directed by professor D’Angelo at the Istituto Neurologico Casimiro Mondino of Pavia. TMS technique is used according to specific guidelines (Rossi et al., 2009).

rTMS within current guidelines poses low risk of adverse effects in BPD and HC groups. The most severe adverse effect is considered nonintentional seizure after rTMS. From many studies that have used TMS, 16 such cases have been reported. Based on the available data, the reported risk of seizures is less than 1 in 1000 for rTMS. Because TMS can have lasting effects on cortical excitability depending on the stimulation parameters (largely frequency and intensity), the seizure risk is related to how the stimulation is applied. High-frequency stimulation can raise cortical excitability and may be unsafe if performed outside the safety guidelines, whereas low-frequency stimulation can reduce cortical excitability (Touge et al., 2001).

Physiological monitoring should minimally involve visual inspection that the muscle twitch from TMS remains limited to the associated body part and seems to immediately follow the stimulus. At the beginning and at the end of the experimental procedure a period of 10–15 min is utilized to observe subjects’ reactions and to answer questions or clarifications about the technique and correlated subjective sensations. Every adverse effect is transient.

A written consent is required and every subject has to fulfill a specific and detailed questionnaire in order to evaluate individual physiological and pathological conditions (such as pregnancy, pace makers, seizures etc.).

### Data Analysis

SPSS for Windows software, version 11.0 (SPSS Inc., Chicago, IL, USA) and Matlab, version 8.5 were used to perform all statistical analyses, and the two-tailed statistical significance level was set at *p* < 0.05, Bonferroni correction was applied if necessary.

To compare the AGN task scores between the different experimental conditions, the Mann-Whitney U test was used since the assumption of normality for the general linear models for repeated measures was not verified; Bonferroni correction for multiple comparison was performed to adjust the level of significance (*p* < 0.013). Spearman’s correlation analyses were used to explore a possible association between clinical variables and neurocognitive test performances.

## Results

### Neuropsychological Performance at Baseline (Pre rTMS)

**Figure 1:**
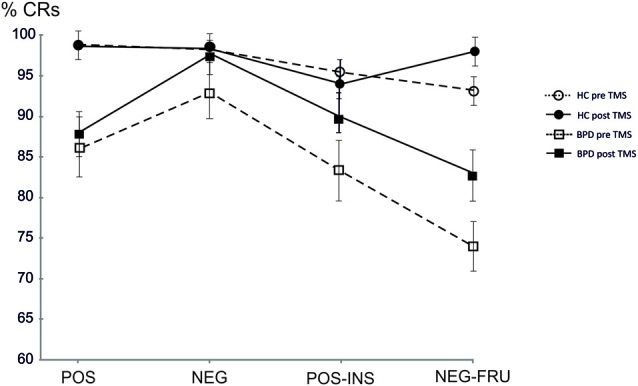
**Mean accuracy at the affective Go/No-Go task (AGN) task in healthy controls (HC) and borderline personality disorder (BPD) groups pre and post repetitive transcranial magnetic stimulation (rTMS) in the four blocks.** HC pre, Mean accuracy in HC pre rTMS; HC post, Mean accuracy in HC post rTMS; BPD pre, Mean accuracy in BPD pre rTMS; BPD post, Mean accuracy in BPD post rTMS; POS, POSITIVE target category words; NEG, NEGATIVE target category words; POS-INS, POSITIVE-INSECTS target category words; NEG-FRU, NEGATIVE-FRUITS target category.

BPD patients performed generally worse than controls in the AGN task (see “Results” Section in Figure 1 and Tables 2–4 below). In particular, BPD mean scores were significantly lower in the final two double category blocks (POS-INS: *p* = 0.011, Mann-Whitney test; NEG-FRU: *p* = 0.0005, Mann-Whitney test). Spearman’s correlation analyses showed that severity levels, as detected with the Zanarini scale, were not correlated with the AGN task scores before or after stimulation. No significant correlations were found between neuropsychological performances and any other variables in BPD and HC groups (Spearman’s correlation analyses, *p* > 0.05).

**Table 2:** **Affective Go/No-Go task (AGN) task performance in HC and BPD groups**.

	HC Mean	BPD Mean
% CRs pre	0.95 ± 0.03	0.85 ± 0.07
% CRs post	0.96 ± 0.01	0.90 ± 0.07
RT pre (ms)	663 ± 65	709 ± 49
RT post (ms)	653 ± 46	705 ± 55
% nCR pre	0.04 ± 0.04	0.14 ± 0.07
% nCR post	0.03 ± 0.01	0.11 ± 0.08

*The table shows the ensemble results of AGN task in HC and BDP groups. HC, Healthy Controls; BPD, Borderline Personality Disorder; % CRs pre/post, number of correct responses pre and post rTMS; RT, Reaction Time pre and post rTMS; % nCRs, number of incorrect responses pre and post rTMS*.

**Table 3:** **AGN task performance pre/post repetitive transcranial magnetic stimulation (rTMS) in HC and BPD groups**.

	Accuracy %			
	HC		BPD	
	Pre	Post	Pre	Post
POS	98 ± 0.03	98 ± 0.03	87 ± 0.15	88 ± 0.14
NEG	97 ± 0.05	97 ± 0.02	93 ± 0.04	96 ± 0.04
POS-INS	94 ± 0.06	96 ± 0.03	83 ± 0.1	91 ± 0.06
NEG-FRU	93 ± 0.05	93 ± 0.05	74 ± 0.12	84 ± 0.12

*The table shows the mean accuracy (% Correct Responses) ± standard deviation (SD) of AGN task in the different blocks in HC and BPD groups. HC Accuracy pre and post, Accuracy of Healthy Controls group; BPD Accuracy pre and post, Accuracy of Borderline Personality Disorder group; POS First block, POSITIVE target category words; NEG Second Block, NEGATIVE target category words; POS-INS Third Block, POSITIVE-INSECTS target category words; NEG-FRU Fourth Block, NEGATIVE-FRUITS target category*.

**Table 4:** **Statistical assessment of comparison of HC and BPD pre and post rTMS in AGN task**.

	Pre rTMS	Post rTMS	Delta Impr.
POS	*p* = 0.037	*p* = 0.059/0.028	*p* = 0.592
NEG	*p* = 0.095	*p* = 0.370/0.626	*p* = 0.243
POS-INS	*p* = 0.011*	*p* = 0.138 /0.110	*p* = 0.062
NEG-FRU	*p* = 0.0005*	*p* = 0.276/0.0707	*p* = 0.024

The table shows the results of the statistical test comparing HC to BDP performance at AGN task in the four blocks (significance level was set at *p* < 0.013 following the Bonferroni correction for multiple comparison and it was marked with *). The neuropsychological AGN performance at baseline (pre rTMS) is compared to mean improvement after rTMS in HC and BPD groups. The AGN task in the two groups was not different in the single category blocks (*p* > 0.013 pre and post rTMS). In the double category blocks of the AGN task, the performance in the two groups was significantly different before rTMS (*p* < 0.013) while became similar after rTMS (*p* > 0.013). POS First block, POSITIVE target category words; NEG Second Block, NEGATIVE target category words; POS-INS Third Block, POSITIVE-INSECTS target category words; NEG-FRU Fourth Block, NEGATIVE-FRUITS target category. Delta impr, difference between mean accuracy post rTMS and pre rTMS.

### Neuropsychological Performance After TMS

After TMS, BPD performance became similar to the HC performance, especially in the last two blocks. The results are summarized in Figure 1 and in the Tables 2–4 (*p* values refer to the comparison between BPD and HC groups in each category block of the AGN task). The improvement was calculated by considering the variation in mean accuracy between pre and post TMS (delta improvement). This variation appears to be different in HC group especially in the two double category blocks.

The HC group showed a non significant improvement in the AGN task after rTMS. The CRs rate moved from 95% (pre rTMS) to 96% (post rTMS). The mean reaction time (RT) changed from 663 ms to 653 ms. BPD group demonstrated a greater but yet not significant improvement in mean accuracy, moved from 85% (pre rTMS) to 90% (post rTMS). The mean RT changed from 709 ms to 705 ms (see Table 2). Considering each block separately, BPD group showed relevant improvement in the final blocks of AGN task, moving from 83% (pre rTMS) to 90% (post rTMS) in the POS-INS block and from 74% to 84% in the NEG-FRU block. In the HC group, mean accuracy during AGN task in the latest two double blocks didn’t change, moving from 93% to 95% in the POS-INS block and remaining at 93% in the NEG-FRU block (see Table 3).

The AGN task in the two groups was not different in the single category blocks (*p* > 0.013 pre and post rTMS). In the double category blocks of the AGN task, the performance in the two groups was significantly different before rTMS (*p* < 0.013) while became similar after rTMS (*p* > 0.013; see Table 4). In the double-category part of the AGN task, BPD patients perform worse before rTMS and approach HC performance after rTMS. Thus, effect of the rTMS appeared to be significant in deficitary cognitive domains while could be considered non influential in physiological conditions.

## Discussion

The central result of this article is that cerebellar rTMS could modulate the response of BPD patients in an AGN task. Given that AGN is normally controlled by the PFC, this result also suggests the presence of prefrontal control deficits in BPD that may be linked to dyscontrolled impulsivity and sustained by anatomical and functional impairment of the DLPFC.

The comparison between accuracy and RTs in HC and BPD subjects suggests an interaction between cognitive demand and inhibitory control in BPD. In particular, the data indicate that the neuropsychological performance at AGN in BPD is especially impaired when cognitive demands are high and require complex associative capacities, as in the final double category blocks of the test. In fact the task in the last two blocks requires a higher working memory load, since the subject must keep in mind both categories to give the CR. Deficits in neuropsychological performance in BPD when working memory demands are high has been demonstrated elsewhere in our previous work (Sala et al., 2009; Lazzaretti et al., 2012). This finding supports the relationship between working memory load and impulsivity, that has been already demonstrated in literature (Hinson et al., 2003).

At baseline, patients performed particularly well in the NEGATIVE single category block (93% of CRs, see Table 2). An increased attentional process in the domain of selective attention when negative stimuli are involved and thus a deficit of inhibition of irrelevant information of aversive nature has been demonstrated in various studies (Domes et al., 2006; Soloff et al., 2015). The inability of borderline patients to disengage attention from negative stimuli may explain the good performance in the block where lack of inhibition of negative stimuli is advantageous (i.e., the target category NEGATIVE) and a worse performance where inhibition of negative stimuli is required (i.e., the block with target category POSITIVE).

The most attracting aspect of these results is that the BPD improvement after cerebellar rTMS emerged when the AGN task became more complex. Before rTMS, BDP patients performed worse than HC in the POS-INS block and in the NEG-FRU block, while after rTMS, the two groups became comparable (see Table 3). Thus cerebellar rTMS demonstrates a relevant effect in the deficitary but not in the preserved cognitive domains. The cerebellum is deeply involved in integration and coordination of different specific stimuli (attention, sensory-motor control, error detection and prediction) and this could be the reason why rTMS effect was more evident in complex associative tasks. We hypothesize that BPD patients have an altered cerebello-thalamo-cortical functional connections resulting in emotional dysregulation and disturbed impulse control. rTMS over the left cerebellum seems to interfere with existing functional connections with a facilitating effect on prefrontal inhibitory control.

The interpretation of this result in terms of neuromodulation is quite complex since there are no preliminary data about the neurophysiological effect of rTMS at 1 Hz on the cerebellum in BPD. It is known that rTMS can modulate cortical excitability in a frequency dependent manner. High frequency rTMS (≥5 Hz) was shown to induce LTP-like effects, whereas low frequency rTMS (≤1 Hz) leads to LTD-like effects (Fitzgerald et al., 2006; Thut and Pascual-Leone, 2010). Empirical data (Oliveri et al., 2005) suggest that low-frequency rTMS trains can produce plastic changes in the cerebellar cortex, similar to those reported for many cerebral areas, including motor, prefrontal and premotor cortex. Indeed, 1 Hz rTMS over cerebellar cortex produced different effects on Intracortical Facilitation (ICF) in three different studies, which may be related to different inter-stimulus intervals chosen in those studies (Fierro et al., 2007; Oliveri et al., 2005).

Recent research has focused on the potential effects of rTMS and tACS on cortical oscillatory activity in a frequency specific manner (Alagapan et al., 2016; Peng and Tang, 2016). Alongside the LPT/LTD mechanisms, rTMS could modify cortical excitability and plasticity inducing modulations of ongoing oscillations, which are deeply involved in functional brain networks. In particular, rTMS in the alpha frequency band seems to vary sensory detection, perception and performance (Klimesch et al., 2003; Thut et al., 2011).

The way by which temporally patterned non invasive stimulations alter cortical oscillatory dynamics is still controversial. In particular, high-frequency rTMS (10 Hz) induces a transient synchronized activity for delta (δ) and theta (θ) rhythms while low-frequency rTMS (1–5 Hz) shows the opposite effect of de-synchronizing low-frequency brain rhythms (Thut et al., 2003; Fuggetta and Noh, 2013).

The mechanistic understanding of underlying changes in brain activity should be further analyzed, in order to create more targeted stimulation designs (Fröhlich, 2015). For example, Farzan et al. (2016) recently demonstrated that iTBS on right lateral CrusI/II and Lobules VIIA/VIIB subregions increased the complexity of brain signal across multiple time-scales in cortical areas corresponding to the network stimulated through cerebellum.

Moreover, it has been demonstrated by MRI that the activity changes induced by rTMS are not restricted to the directly stimulated area, but involve functionally connected remote areas (Paus et al., 2001; Strafella et al., 2001). Such cross-modal plasticity-like effects may be transferred via direct cortico-cortical connections, indirectly via multi-sensory association areas, or via subcortical interplay at the thalamic level. Thus, it is possible that cerebellar rTMS on the posterior lateral lobe, a “cognitive” part of the cerebellum, has an effect on the PFC through the cerebello-thalamo-cortical fiber tracts (Palesi et al., 2015). Our results confirm that low-frequency rTMS has a facilitatory effect on the PFC, as hypothesized in the “Introduction” Section.

The neurophysiological mechanisms are not easily predictable though, and interpretation of reported results is even more complex when considering indirect measures of cortical excitability such as a neurocognitive performance instead of electromyographic (EMG) activity. It is possible that low-frequency rTMS induced a transient depression of Purkinje cell excitability, thereby disinhibiting cells in the dentate nucleus, the site of origin of the output fibers directed toward the cereberal cortex. Dentate cell disihinibition could have resulted in a facilitation of the thalamus and PFC.

It is known that “these cortico-cerebellar loops are involved in the identification of errors and novelty (sensory prediction) and can trigger automatic corrections, promote learning and redirect attention” (D’Angelo and Casali, 2012). Accordingly, cerebellar alterations affecting the cerebello-cortical loops may lead not only to the well known motor abnormalities but also to behavioral, cognitive and affective alterations, usually correlated to frontal regions and limbic areas. The cerebellum is fundamental for contextualizing external and internal stimuli and coordinating their spatio-temporal evolution, generating coherent ensemble activities. Therefore, dysfunction of the cerebellar circuits and of information reentry toward the frontal and parietal cortex may contribute to preventing the formation of coherent and contextualized behaviors. Additionally, the cerebellum is critical for revealing differences between predictions elaborated by the cortex and the stimuli conveyed by the senses. Thus, dysfunction of the cortico-cerebellar circuits may affect the detection of novelty and impair attention switching (D’Angelo, 2011). These complex functions (sensory prediction and novelty detection) seem to be impaired in BPD patients and are widely described by clinicians. For this reason a study focused on cortico-cerebellar projections’ functionality could help us to clarify the neurophysiological correlates of BPD patients.

To the best of our knowledge, this could be the first study investigating if BPD patients show differences in cerebellar rTMS parameters compared with HC.

### Limitations and Perspectives

Some limitations of this study need to be mentioned. First, the small sample size may not have provided enough statistical power to detect subtle abnormalities and changements. This is a pilot study, recently defined (Leon et al., 2011) as a requisite initial step in exploring a novel intervention or an innovative application of an intervention. Our results inform feasibility and identify integrations needed in future design of a larger, ensuing hypothesis testing study. Our main interest was to explore cerebellar involvement in a psychiatric syndrome that has always been associated to prefrontal, hippocampal and limbic dysfunctions.

According to the characteristics of a pilot study, we did not provide a preliminary sample size determination. The sample size is relatively modest, although comparable to previous neuro- anatomical studies in this field (Monarch et al., 2004). Second, the majority of BPD patients had other comorbid diagnoses in the past few years, as it is often seen in the real world (Skodol et al., 2002a,b). Therefore, excluding subjects with Axis I comorbidity would create a non-representative BPD patients sample that could ultimately limit the generalizability of the findings.

BPD patients recruited in this study were taking psychopharmacological therapy at the time of testing (anti-depressants, mood-stabilizer and antipsychotics). Medications and pharmacological status are potential confounders since neurotropic substances interact with neurotransmitter systems involved in cortex plasticity. Nonetheless, since our main findings deal with specific section of a neurocognitive task in comparison and not with a general effect of rTMS on plasticity, we could consider them still significant.

This study is going to be extended, recruiting a larger sample size and then providing a sham control group, in order to evaluate aspecific placebo effects of the experimental procedure. As far as the impact of learning in the neuropsychological task is concerned, we tried to limit significant learning effects between the first and the second neuropsychological test by pre-training subjects with AGN to improve their skills. The high and almost constant performance of healthy subjects (between 95% and 100%) across different sub-tasks suggests that the procedure as a whole was effective, nonetheless the impact of learning may be further investigated through a rigorous behavioral assessment. In our study, we didn’t counterbalance the task blocks across subjects. This was because we wanted to preserve an increasing difficulty level among blocks and we already controlled for the learning effect through blocks by mean of a short preliminary training. It is possible that performance of BPD patients in the last two blocks was influenced also by a “fatigue effect” besides the higher complexity of the task. Nevertheless, this doesn’t affect our main finding of a significant improvement after TMS in the last two blocks for BPD patients that could not be dependent on any order effect. This pilot study has been useful in exploring an innovative application of the rTMS intervention in a clinical population characterized by little compliance in experimental procedures. The obtained results confirm the feasibility of this approach and encourage large scale studies.

The present study has to be extended in order to deepen the physiological nature of the changes that occur in the PFC after cerebellar rTMS. A limit of this and of previous studies, is the anatomical spatial localization of this brain region, based on scalp coordinates rather than on the use of neuronavigation systems that improve the connection between the coil positioning on the scalp and the underlying brain structure. Further studies, aimed to analyze cerebro-cerebellar interactions anatomically and not only functionally, should utilize these systems. Moreover we are going to integrate the rTMS procedure with EEG and fMRI registrations during the same neuropsychological paradigm, in order to identify circuits involved in impulsivity and prefrontal inhibitory control. fMRI will allow a precise identification of the cerebellar area of interest.

### Conclusion

These results lead to the hypothesis that BPD patients have altered cerebello-thalamo-cortical connections resulting in emotional dysregulation and disturbed impulse control. This is in line with recent anatomical and clinical evidence showing that the cerebellum, primarily considered as a motor control structure, is also involved in higher cognitive functions and behavioral changes, such as impulsive behavior. The rTMS over the left cerebellum could have interfered with existing functional connections exerting a facilitating effect on prefrontal inhibitory control in complex cognitive domains. The present study needs to be extended in order to give more insight into the physiological nature of the changes that occur in the PFC after rTMS of the left cerebellum.

## Author Contributions

GZDV wrote the protocol, performed patients selection and diagnostic evaluation, performed TMS tests and wrote the body of the article. RM contributed to patient selection and text writing. JM performed TMS tests and data analysis. NC engineered the stimulation/recording system and the data analysis programs. DB contributed to healthy control selection. EC and FB coordinated the patients facility and diagnostic operations. EDA coordinated the work and ethical committee application, supervised the neurophysiological part and the final version of the text.

## Conflict of Interest Statement

The authors declare that the research was conducted in the absence of any commercial or financial relationships that could be construed as a potential conflict of interest.
